# Venous Thromboembolism Prophylaxis Use by Pediatric Orthopedic Surgeons in Canada for the Pediatric Orthopedic Surgical Population

**DOI:** 10.7759/cureus.29933

**Published:** 2022-10-05

**Authors:** Jenna Curwin, Wyatt MacNevin, Ron El-Hawary, Ketan Kulkarni

**Affiliations:** 1 Pediatric Hematology and Oncology, Dalhousie University, Halifax, CAN; 2 Urology, Dalhousie University, Halifax, CAN; 3 Orthopedics, Izaak Walton Killam (IWK) Health Centre, Halifax, CAN; 4 Hematology and Oncology, Izaak Walton Killam (IWK) Health Centre, Halifax, CAN

**Keywords:** screening protocol, venous thromboembolism (vte), pediatric orthopedic surgery, perioperative risk factors, venous thromboembolism prophylaxis

## Abstract

Introduction

A novel pediatric venous thromboembolism (VTE) screening tool was implemented in 2016 at the Izaak Walton Killam (IWK) Health Centre, which safely reduced the use of thromboprophylaxis by 47.9% with no increase in VTE in the pediatric orthopedic surgical population (POSP). There is presently no data on the current practices or protocols for VTE prophylaxis for POSP in Canada. The present survey was designed to assess current practices regarding VTE prophylaxis for POSP in Canada.

Methods

After research ethics board (REB) approval, a 22-question survey was administered electronically to all Canadian Pediatric Orthopedic Group (CPOG) members. The survey contained questions on respondent demographics and background, current VTE prophylaxis practices and experiences including indications for prophylaxis, the existence of VTE protocols, and interest in utilizing VTE protocols. Descriptive statistical analyses and analysis of variance (ANOVA) were completed on the survey responses.

Results

Of the 100 CPOG members, 49 (49%) responded. Most respondents (51%, n=25/49) practice in Central Canada, 39% (n=19/49) practice in Western Canadian provinces, and a smaller portion practice in Atlantic Canada (10%, n=5/49). Of the respondents, 43% (n=21/49) indicated that they use pharmacologic VTE prophylaxis in their practice, and 93% (n=27/29) stated that specific risk factors are indications of initiating pharmacologic VTE prophylaxis. Additionally, 57% (n=16/28) did not have a defined protocol for VTE prophylaxis, and 18% (n=5/28) were uncertain if they do. Of the respondents, 85% (n=22/26) were open to utilizing a VTE prophylaxis screening tool, and 12% (n=3/26) were uncertain if they would be.

Conclusion

This study has demonstrated that a uniform protocol for VTE prophylaxis does not exist in most Canadian centers, despite its need. There is nationwide interest in adopting a perioperative VTE prophylaxis screening tool to optimize pharmacologic thromboprophylaxis use in POSP. The goal of future research is the national implementation and standardization of such a screening tool through collaboration in a multicenter study.

## Introduction

Venous thromboembolism (VTE) is a hematologic condition when a blood clot forms and presents as a deep vein thrombosis (DVT) and/or a pulmonary embolism (PE) [1]. VTE is a leading cause of morbidity and mortality among surgical patients, and the incidence of VTE among at-risk pediatric surgical populations has been increasing as medical techniques and knowledge improve [2]. The incidence of VTE in the pediatric orthopedic surgical population (POSP) is reported to be 0.1%-0.2% [3-5]. Despite a low prevalence in this population, thromboprophylaxis is relevant to pediatric orthopedic surgeons because of the association of VTE with morbidity and mortality [1].

The low incidence of VTE, inherent risks of thromboprophylaxis (such as bleeding complications), and financial considerations are all reasons why universal thromboprophylaxis cannot be recommended in this population. In 2016, at the Izaak Walton Killam (IWK) Hospital for Children, a screening tool for all perioperative pediatric orthopedic surgical populations (POSP) was developed [2]. Using the screening tool, patients are given a VTE risk score, and patients identified as high risk require a consultation with the Department of Hematology to confirm the use of thromboprophylaxis based on patient and surgical risk factors. The implementation of this screening tool at the IWK has been shown to safely reduce thromboprophylaxis use by 47.9% [2].

Currently, minimal literature exists on the use of thromboprophylaxis and VTE prevention protocols for POSP in Canada. Data is not clear on the availability and use of such tools, and a national consensus does not exist [1]. To determine if national implementation and standardization of such a screening tool can be achieved, knowledge on willingness to adopt a tool needs to be established.

This study surveyed pediatric orthopedic surgeons across Canada on their perspectives on VTE prophylaxis and the use of screening tools for the pediatric orthopedic surgical population at their respective institutions.

This article was previously presented as a poster at the International Society on Thrombosis and Haemostasis (ISTH) Congress in London, UK, on July 12, 2022. This article was previously presented as a meeting abstract at the Department of Pediatrics Trainee Research Day at Dalhousie University on June 13, 2022, and at the Canadian Pediatric Thrombosis and Hemostasis Network (CPTHN) 2022 annual general meeting on May 6, 2022.

## Materials and methods

A 19-question survey on VTE prophylaxis use by Canadian pediatric orthopedic surgeons was designed based on similar surveys implemented in the USA [1,6]. The survey was developed after a thorough literature review, consultation with national and international experts, and using a modified nominal and consensus development conference (serial meetings) method for reaching a consensus [7,8]. Survey questions were developed and reviewed for consistency, reliability, and validity. The survey was initially piloted locally to assess any inconsistencies and refined accordingly. Discussions with members of the Canadian Pediatric Orthopedic Group (CPOG) indicated support for the development of the survey to determine the current state of perioperative VTE prophylaxis for the pediatric orthopedic surgical population in Canada. The CPOG is a research group that provides a collaborative research forum for orthopedic surgeons and allied healthcare professionals practicing in Canada and is the largest pediatric orthopedic organization in Canada.

This survey included questions about the respondent’s demographic and background information (e.g., province/territory of practice, practice location, and annual surgical volume), their current VTE prophylaxis practices and experiences (e.g., indications for use and complications), VTE protocol use at their institution, and interest in collaborating on national VTE prophylaxis initiatives. The full list of survey questions can be found in the Appendices.

This study received research ethics board (REB) approval through the Isaac Walton Killam (IWK) Health Centre in Halifax, Nova Scotia, and the REB file number is 1026057. The survey was distributed to all CPOG members. After obtaining research ethics board approval, the survey was distributed to all Canadian Pediatric Orthopedic Group (CPOG) members. The survey was delivered using Dalhousie University’s online Opinio survey platform. After the initial request to complete the survey, reminder emails were distributed two, four, and eight weeks after initial distribution. There was no compensation for completing the survey.

The survey responses were analyzed using the Statistical Package for the Social Sciences (SPSS) software version 27 (IBM Corp., Armonk, NY, USA). Descriptive statistical analyses and analysis of variance (ANOVA) were performed. Provinces/territories were grouped on a regional basis.

## Results

Demographics

A 49% (n=49/100) response rate was obtained when surveying the CPOG members. The majority of the respondents (51%) practice in Central Canada (Ontario and Quebec), 39% practice in Western Canadian provinces (Manitoba, Saskatchewan, Alberta, British Columbia, and the Yukon and Northwest Territories), and 10% from Atlantic Canada (New Brunswick, Nova Scotia, Prince Edward Island, and Newfoundland and Labrador). More respondents practice in a non-academic center (55%) compared to a university hospital/academic institution (45%). At most centers (54%), over 250 procedures had been performed in the past 12 months. Additionally, 8% completed 200-249 procedures in the past 12 months, 4% completed 150-199 procedures, and an equal amount (17%) completed 100-149 and 50-99 procedures.

VTE prophylaxis use in Canada

Of the respondents, 43% (n=21/49) indicated that they use pharmacologic VTE prophylaxis in their practice. From this point on in the survey, respondents who indicated that they do not use pharmacologic VTE prophylaxis were not required to complete the remainder of the survey. Of the original 49 respondents, 30 completed the remainder of most questions.

When asked about specific indications for initiating pharmacologic VTE prophylaxis, 67% (n=20/30) stated that a specific age is an indication, 63% (n=17/27) said specific diagnostic/procedural categories are indications, and 93% (n=27/29) stated specific risk factors are indications (Table 1). Of those who specified age as an indication, one respondent specified the age as 12 years old, three respondents said 14, three respondents said 16, and two respondents said 18. Additionally, one respondent specified that all patients in the age range of 14-18 years old are administered thromboprophylaxis.

**Table 1:** Specific risk factor indications for initiating pharmacologic venous thromboembolism prophylaxis for the pediatric orthopedic surgical population. ICU: intensive care unit

Specific risk factor	Frequency (n=27)
History of thrombophilia	24
Medications (i.e., oral contraceptives)	14
Oncologic diagnosis	12
Anatomic considerations of pathology	7
Duration of surgery	6
Paralysis	1
Trauma in the ICU	1
Reduced weight-bearing status	1

For all questions regarding pharmacologic VTE prophylaxis indications, 12 respondents stated trauma and four stated spine surgery as specific diagnostic categories that are indicative of receiving pharmacologic VTE prophylaxis. Of the respondents who said that specific risk factors are an indication for pharmacologic VTE prophylaxis, a history of thrombophilia was the most common risk factor (88%, n=24/27), followed by medications (52%, n=14/27), oncologic diagnosis (44%, n=12/27), anatomic considerations (26%, n=7/27), and surgery duration (22%, n=6/27).

Of the respondents, 79% (n=23/29) do not have a hospital-initiated method for initiating pharmacologic VTE prophylaxis based on age, one respondent was uncertain if their hospital does, and one stated that the age of 18 years was used for initiating VTE prophylaxis. In terms of agents for pharmacologic VTE prophylaxis, 20 respondents said that they use low-molecular-weight heparin, and five respondents stated that they use acetylsalicylic acid (ASA).

With regard to mechanical VTE prophylaxis in the pediatric orthopedic surgical population, of the original 49 respondents, 43% (n=21/49) do not use mechanical VTE prophylaxis, and 47% (n=23/49) only use it for individual patients who the surgeon deems high risk. Of the respondents, 6% (n=3/49) use mechanical VTE prophylaxis less than 25% of the time, and one respondent uses it 50%-74% of the time. When mechanical VTE prophylaxis agents are used, 11 (48%, n=11/23) respondents use sequential compression devices, and 11 use thromboembolic deterrent stockings.

VTE and VTE prophylaxis complications

In the last five years, 73% (n=22/30) had not experienced a complication related to VTE, and 27% (n=8/30) had experienced a complication as follows: (i) a 13-year-old with a Taylor spatial frame with minimal open surgery who developed a PE, (ii) anterior cruciate ligament reconstruction for a patient on an oral contraceptive pill who developed DVT, (iii) DVT in tibia fracture patient who was completely sedentary for weeks, (iv) DVT in a high body mass index (BMI) teen patient after complicated spine surgery, (v) one patient with axillary vein thrombosis post-trauma, and (vi) PE in a 16-year-old with high BMI who underwent femur fracture revision nailing. One respondent (n=1/29) noted a complication of easy bruising and prolonged wound drainage related to pharmacologic and/or mechanical VTE prophylaxis in the last five years. Of the respondents, 28/29 (97%) denied any associated complications with mechanical or pharmacologic VTE prophylaxis in the last five years.

Current prophylaxis protocol use

When asked if the hospital at which they perform pediatric orthopedic surgery has a defined protocol for VTE prophylaxis, 25% (n=7/28) said yes, 57% said no, and 18% were uncertain. The province where the respondents practice was not associated with having a defined protocol for VTE prophylaxis (p=0.38). When queried on how their protocols are initiated, the majority responded that their protocols are initiated based on similar indications as shown in Table 1, such as a personal history of thrombophilia and oral contraceptive use. When respondents were asked about following the VTE prophylaxis protocol implemented at their institution, 32% (n=7/22) reported following it 100% of the time (except for patients at high risk of complications), 55% (n=12/22) unintentionally do not always follow their VTE protocols at their institution (e.g., no systems in place to trigger or monitor implementation of protocol), and 14% (n=3/22) do not always follow the protocol when specific indications are met.

If their hospital does not have a defined protocol for VTE prophylaxis, the respondents were asked if they would be interested in utilizing a VTE prophylaxis screening tool, and 85% (n=26) responded yes, 12% indicated that they were uncertain, and only one respondent responded no.

## Discussion

This study has demonstrated nationwide interest in adopting a perioperative VTE prophylaxis screening tool to optimize the use of pharmacologic thromboprophylaxis in the pediatric orthopedic surgical population (POSP). Although the prevalence of VTE in the pediatric orthopedic surgical population is low, thromboprophylaxis in this population is important due to the association of VTE with morbidity and mortality [1]. Guzman et al. analyzed the 2012 Kids Inpatient Database and found an overall baseline mortality rate of 0.53%, which increased to 0.9% for children with a fracture and further to 1.8% in children with associated diagnoses of VTE and a fracture [9]. Additionally, postoperative VTE in the pediatric orthopedic surgical population has been shown to increase the length of stay and lead to considerable morbidity [10,11]. Due to this, it is important for VTE thromboprophylaxis to be optimized.

The risk factors for VTE in the adult population have been clearly recognized, and there are associated prophylactic protocols that have been implemented. However, the same is not true of the pediatric population in Canada [7]. Identifying which patients are at the highest risk of VTE is crucial for optimizing the use of VTE prophylaxis. In the USA, pediatric prophylactic guidelines have been successfully implemented to identify high-risk pediatric populations [12]. In patients with no identifiable risk factors, thromboprophylaxis may not be necessary [10]. The risk factors for VTE in the pediatric orthopedic surgical population have been studied to include increased age, obesity, malignancy, major trauma, coagulopathy, and length of stay [9,13,14]. These highlighted risk factors can be compared with those described in this study as specific risk factor indications for initiating pharmacologic VTE prophylaxis. Specifically, a history of thrombophilia, medications such as an oral contraceptive, oncologic diagnosis, age, trauma, and spine surgery were all reflected in the survey responses. Despite agreement on significant VTE risk factors within this population, most respondents (75%, n=21/28) do not currently have a protocol in place at their institution for VTE risk screening, or they are uncertain if one exists. Only 32% (n=7/22) surveyed follow their protocol 100% of the time if one does exist. This supports the need for the implementation of a uniform, accessible bedside screening tool. This demonstrates that although nearly half (43%, n=21/49) of the respondents indicated that they use pharmacologic prophylaxis in their practice, it is used by physician preference in a non-algorithmic way in Canada. This is problematic as it is not an evidence-based, standardized approach, and therefore, thromboprophylaxis may be administered to patients who do not require it, and high-risk patients may be missed.

This survey has also identified a need for education on the purpose of prophylaxis and the appropriate agents for use. Of the respondents, 17% (n=5/30) indicated that they use ASA for pharmacologic VTE prophylaxis. There has been no evidence that ASA is as effective as low-molecular-weight heparins or novel oral anticoagulants for VTE thromboprophylaxis [15]. The use of a standardized screening tool would help facilitate discussions with hematology for appropriate prescribing.

A strength of the present study is that it is a Canadian-wide initiative with respondents from different provinces and institutions across Canada with approximately 50% of the Canadian Pediatric Orthopedic Surgical Group (CPOG) participating. The survey was highlighted in the national journal club and the annual meeting for the CPOG to optimize awareness and response rates. This study builds on a protocol that was developed at the IWK and tested for several years to prove its efficacy [2].

The limitations of this study include known limitations of survey methodology. Surveys are subject to response bias, which is when individual factors and/or thought processes that take place during the process of responding to surveys affect the way responses are provided [16]. Response bias leads to a nonrandom deviation of the answers from the true answer. Surveys are also subject to missing data when certain questions are left unanswered by respondents [17]. This study addresses potential interest in collaborating in a multicenter study utilizing a perioperative screening tool, but the study is limited in the fact that it did not include questions on the logistical challenges centers face in implementing such a protocol. Further studies are needed to assess the same.

This current study has demonstrated significant interest in adopting a uniform approach to VTE prophylaxis in this population. Future studies are focused on implementing a standardized VTE thromboprophylaxis screening tool as the basis of a multicenter study on the efficacy of a Canadian VTE screening tool.

## Conclusions

In conclusion, roughly half of the respondents use pharmacologic VTE prophylaxis in their practice, yet the majority do not have a protocol in place or are unfamiliar with a protocol at their institution. This variation in practice could potentially put the pediatric orthopedic surgical population at risk for adverse events with respect to VTE. Most respondents indicated specific risk factors as an indication for initiating prophylaxis, and those risk factors reflect those published in the literature. The majority of respondents would be open to utilizing a VTE risk screening tool. This study highlights the need for further research to work toward implementing a uniform VTE prophylaxis perioperative screening tool. This tool would enable nationwide identification of patients within this population who are at higher risk for VTE and reduce the unnecessary use of pharmacologic thromboprophylaxis and plausibly the occurrence of VTE.

## Appendices

Survey questions

Demographic and Background Information

1.   In which Canadian province or territory do you practice?

a.   Alberta

b.   British Columbia

c.   Manitoba

d.   New Brunswick

e.   Newfoundland and Labrador

f.    Nova Scotia

g.   Ontario

h.   Prince Edward Island

i.    Quebec

j.    Saskatchewan

k.   Northwest Territories

l.    Nunavut

m.  Yukon

2.   Where do you practice orthopedics? Choose all that apply.

a.   Community hospital

b.   Children’s hospital

c.   University hospital/academic center (adult/children)

d.   Other, please specify: ________

3.   Approximately how many surgeries on children (under 18 years) did your center perform in the last 12 months?

a.   <50

b.   50-99

c.   100-149

d.   150-199

e.   200-249

f.    ≥250

4.   Do you use mechanical VTE prophylaxis in addition to pharmacologic prophylaxis?

a.   100%

b.   75%-99%

c.   50%-74%

d.   25%-49%

e.   <25%

f.    I only use mechanical VTE prophylaxis for patients who are at high risk.

g.   I do not use mechanical VTE prophylaxis.

5.   Do you use pharmacologic VTE prophylaxis in your practice?

a.   Yes (Please complete the following questions.)

b.   No (Thank you! You do not need to complete the rest of the survey.)

Current VTE Prophylaxis Practices and Experience

6.   What are your indications for pharmacologic VTE prophylaxis for pediatric surgical patients? Choose all that apply from the following set of questions.

a.   Specific age indications; if yes, please specify: _________

b.   Specific diagnostic/procedural categories (N/A, trauma, spine surgery, other); if other, please specify: _________

c.   Specific risk factors (N/A, oncologic diagnoses, anatomic considerations of the pathology, history of coagulopathy, medications (e.g., birth control pill), duration of surgery, other); if other, please specify: _________

d.   Other, please specify: _________

7.   Does your hospital have an age limit beyond which all patients receive pharmacologic VTE prophylaxis once admitted?

a.   Yes (please specify the age: _______)

b.   No

c.   Uncertain

8.   What agents of pharmacologic VTE prophylaxis do you use? Choose all that apply.

a.   I do not use pharmacologic VTE prophylaxis.

b.   ASA (aspirin)

c.   Low-molecular-weight heparin (Lovenox/enoxaparin)

d.   Rivaroxaban

e.   Apixaban

f.    Synthetic heparin (fondaparinux)

g.   Other, please specify: _________

9.   What agents of mechanical VTE prophylaxis do you use? Please check all that apply.

a.   Compression device

b.   Thromboembolic deterrent stockings (TEDs)

c.   Other, please specify: _________

10.   Have you had a pediatric patient experience a complication related to VTE in your practice in the last five years?

a.   Yes - if so, how many patients and describe the complication(s): _________

b.   No

11. Have you had a patient of yours experience a complication related to pharmacologic and/or mechanical VTE prophylaxis in the last 5 years?

a.   Yes; if so, how many patients and describe the complication(s): _________

b.   No

Existence of VTE Protocols and Details

12.   Does your hospital where you perform surgery on children (under 18 years) have a defined protocol for VTE prophylaxis?

a.   Yes

b.   No

c.   Uncertain

13.   If your hospital where you perform surgery on children does NOT have a defined protocol for VTE prophylaxis, would you be open to utilizing a VTE prophylaxis screening tool?

a.   Yes

b.   No

c.   Uncertain

14. How is your VTE prophylaxis program initiated? Choose all that apply.

a.   N/A

b.   Same indications reported in question 6

c.   Based on diagnosis

d.   Based on risk factors

e.   Based on surgery duration

f.    Age

g.   Unknown

h.   Other, please specify: _________

15.   If your VTE prophylaxis is initiated (based on diagnosis). Choose all that apply.

a.   N/A

b.   Trauma

c.   Spine surgery

d.   Other, please specify: _________

16.    If your VTE prophylaxis is initiated (based on risk factors).

a.   N/A

b.   Please specify: _________

17.   If your VTE prophylaxis is initiated (based on surgery duration).

a.   N/A

b.   Please specify (i.e., >1 hour): _________

18.   Do you follow that protocol?

a.   I follow the protocol 100% of the time (except for patients with a high risk of complications).

b.   I do not always follow the protocol: unintentionally (e.g., no systems in place to trigger or monitor implementation of protocol).

c.   I do not always follow the protocol: specific indications for not following the protocol.

19.   What is the purpose of this research study?

Open text:
